# Intelligent design: stablecoins (in)stability and collateral during market turbulence

**DOI:** 10.1186/s40854-023-00492-4

**Published:** 2023-05-06

**Authors:** Riccardo De Blasis, Luca Galati, Alexander Webb, Robert I. Webb

**Affiliations:** 1 Department of Management, Marche Polytechnic University, Via Lodovico Menicucci 6, 60121 Ancona, AN Italy; 2 School of Business, University of Wollongong, Northfields Ave, 2522 Wollongong, NSW Australia; 3 Derivatives Markets Research Centre, Rozetta Institute, 55 Harrington St, 2000 Sydney, NSW Australia; 4 Department of Economics, University of Molise, Via Francesco De Sanctis, 86100 Campobasso, CB Italy; 5 McIntire School of Commerce, University of Virginia, 140 Hospital Drive, 22903 Charlottesville, VA USA

**Keywords:** Stablecoins, Herding, Information cascades, Volatility spillovers, Market crashes, Financial contagion, D47, F31, F61, G14, G41

## Abstract

How does stablecoin design affect market behavior during turbulent periods? Stablecoins attempt to maintain a “stable” peg to the US dollar, but do so with widely varying structural designs. The spectacular collapse of the TerraUSD (UST) stablecoin and the linked Terra (LUNA) token in May 2022 precipitated a series of reactions across major stablecoins, with some experiencing a fall in value and others gaining value. Using a Baba, Engle, Kraft and Kroner (1990) (BEKK) model, we examine the reaction to this exogenous shock and find significant contagion effects from the UST collapse, likely partially due to herding behavior among traders. We test the varying reactions among stablecoins and find that stablecoin design differences affect the direction, magnitude, and duration of the response to shocks. We discuss the implications for stablecoin developers, exchanges, traders, and regulators.

## Introduction

Since the release of the Bitcoin white paper in 2008, cryptocurrencies have attracted controversy and interest in the financial literature (i.e. Nakamoto 2008). Of the top five cryptocurrencies by trading volume, three are stablecoins, two of the top five by market capitalization are stablecoins. Stablecoins are designed to maintain a “stable” peg to another financial asset, usually the US dollar. They play a crucial role in the market by allowing traders to store value in US equivalents. Stablecoins, particularly Tether which is the third largest cryptocurrency by market value and by far the largest in terms of volume,^1^ may be used as a safe haven for Bitcoin investors (Baur and Hoang 2021). However, stablecoins sometimes trade at a premium to the underlying asset they mimic because of the high fees to trade cryptocurrencies in US dollars, the difficulty of using US dollars on cryptocurrency exchanges, as well as the speed and ease of transferring stablecoins between exchanges (Lyons and Viswanath-Natraj 2020).^2^ Different stablecoins attempt the same goal—maintaining a $1 peg—but with vastly different structural designs and transparency levels. The recent collapse of the TerraUSD (UST) stablecoin and linked Terra (LUNA)^3^ token in May 2022 brought down a $60 billion ecosystem, making this exogenous shock an important natural opportunity for study.

At approximately midnight (UTC time) on May 9, 2022, UST began to experience what would soon become a substantial collapse. By the early hours of May 10, 2022, UST had unambiguously lost its peg with the dollar, trading at 98 cents at 2 a.m. (UTC), 90 cents at 8 a.m. (UTC), but only 79 cents by 9 a.m. (UTC). The impact of the decline in UST was observed with a delay in other cryptocurrency stablecoins. Around the time of the crash, UST had a market capitalization of $18 billion,^4^ valuing it similarly to U.S. corporations such as Best Buy or Clorox. During the days surrounding this period of market turmoil, other stablecoins experienced significant price deviations from their $1 peg. Tether, for example, dropped to 97 cents at around 4 p.m. (UTC) on May 12, 2022, while BUSD rose to 1.0149 and USDC rose to 1.01. In contrast, DAI experienced small fluctuations around $1. Although all the above-mentioned stablecoins aim to maintain a stable $1 peg, they experienced vastly different price behaviors, with some trading at a premium and others at a discount during the crash. This study examines the period before and after the collapse of stablecoin UST on May 9, 2022, tests the extent to which this market crash impacted other major digital assets, and investigates the causes of the hypothesized contagion. In particular, we examine whether differences across stablecoins in the mechanism used to maintain the peg help explain the differences in the magnitude, direction, and duration of their response to the UST stablecoin crash.

Answering this question is important because the May 2022 collapse of UST, alongside the resultant volatility in multiple important stablecoins, demonstrated the fragility of algorithmic stablecoins and the importance of credible collateral for stablecoins linked to fiat currencies. We use intraday data and a multivariate Baba, Engle, Kraft, and Kroner (1990) (BEKK) model over a sample period of 40 days surrounding the UST crash on the 9th of May 2022, to test how differences in stablecoin designs affect trader behavior and market reactions and investigate the causes of spillover effects in the cryptocurrency markets. Previous studies have also used this method to test whether contagion effects are due to herding behavior (for example Corsetti et al. 2005; Boyer et al. 2006; Chiang et al. 2007; Syllignakis and Kouretas 2011). Indeed, we find evidence of contagion effects across all the cryptocurrency and stablecoins analyzed, with some signs of herding behavior by traders after an information cascade that is apparent in the subsequent event study analysis. Deviations from the $1 peg document how traders “vote with their feet” by moving in or out of various stablecoins. Finally, we demonstrate how smaller market players can cause financial contagion, which infects larger players and finally feeds back to the market as a whole.

We contribute to the literature by investigating financial contagion in cryptocurrency markets during turbulent periods such as the UST stablecoin crash. Additionally, to the best of our knowledge, this study is the first to provide implications for stablecoin design, trader behavior, and contagion effects during the stablecoin markets crisis, which is useful for academics, practitioners, and policymakers interested in the potential destabilizing risk arising from the cryptocurrency ecosystem. The originality of this study lies in its examination of a unique exogenous event, the largest collapse in stablecoin markets to date, using proprietary data.

We also extend previous research on the effects of volatility spillovers in cryptocurrency markets to stablecoin markets. Although previous research has attempted to investigate volatility spillover effects between Bitcoin and stablecoins (Hoang and Baur 2021; Grobys et al. 2021), and between stablecoins only (Thanh et al. 2022), to the best of our knowledge, a comprehensive investigation of the magnitude, direction, and duration of the response to stablecoin price movements is yet to be conducted. This study fills this gap and extends previous research on volatility spillover across stablecoins by testing whether differences in their underlying design affect market behavior. Moreover, this study investigates possible herding behaviors in cryptocurrency crashes, as the bubbles in Haykir and Yagli (2022), and tests the information cascade effects, as Tse and Hackard (2006) do in different US markets, precipitated by the UST collapse on other stablecoins market activities, which enable us to make an additional contribution to the literature.

## Literature review

Cryptocurrencies can be considered as privately produced money. However, the idea that money should be decentralized and privately produced is not new. Hayek (1976) argues for the denationalization of money, claiming that money, like other aspects of a capitalist economy, would be most efficiently provided through open competition. He also argues that, by definition, a monopoly cannot efficiently balance supply and that the removal of the government’s monopoly over money would prevent politician-led inflation and other destabilizing state-led interference with currencies. Interestingly, the first Bitcoin block mined contains a message criticizing government bailouts of the financial system; some see cryptocurrencies as an alternative to government-issued currencies.

Not surprisingly, given that stablecoins are a relatively recent innovation, the literature on their stability during turbulent periods is limited. The collapse of the UST resembles other situations in which panic occurs. Indeed, if the research question is viewed more broadly as instances in which pegs in financial markets are broken during turbulent markets, there is related literature on foreign exchange, money market mutual funds, and bank runs. For instance, the collapse of TerraUSD bears some similarities to a run on the Primary Reserve money mutual fund in the wake of the Lehman Brothers’ bankruptcy filing on September 15, 2008. Like the collapse of the Primary Reserve money market mutual fund on September 16, 2008, amid fears that the fund held a substantial amount of potentially worthless Lehman Brothers short-term debt, the collapse of TerraUSD started with “breaking the buck.” Unlike in the Primary Reserve money market mutual fund case, the causes that triggered TerraUSD’s collapse remain obscure to the public. Additionally, unlike the Primary Reserve money market mutual fund collapse, the Federal Reserve and US Treasury did not rush in to guarantee the stability of stablecoins in seeming trouble.^5^ Similar problems can arise in foreign exchange markets. For instance, the Argentine Peso was convertible to the US Dollar on a 1:1 basis under a “hard peg” for the period from April 1991 until January 6, 2002, when the peg broke and the Peso was allowed to float. De La Torre et al. (2003) examine the causes of the sudden failure of the hard peg of Argentina’s Peso to the US Dollar. Hanke and Schuler (2002) argue that the essential reason for the failure was that Argentina did not employ a true currency board system. The commonality in both examples is that fear that the peg will not hold sparks the type of behavior typically observed during a bank run.

A large body of literature has examined the effects of financial markets contagion in periods of crises. Many studies have analyzed the Global Financial Crisis (e.g. Baur 2012; Fry-McKibbin et al. 2014; Kenourgios and Dimitriou 2015), with some focusing on emerging markets (Celık 2012; Boubaker et al. 2016), Asian markets (Yiu et al. 2010), European markets (Syllignakis and Kouretas 2011), or foreign exchange markets (Ding and Vo 2012) with bond, equity, and commodity markets (Diebold and Yilmaz 2012). Others have instead investigated crises such as the Covid-19 Pandemic (Akhtaruzzaman et al. 2021; Uddin et al. 2022), or both the Global Financial Crisis and the Covid-19 Pandemic (Nguyen et al. 2022). Overall, all the aforementioned studies find that during periods of market turmoil or economic shocks, financial markets react by spreading volatility effects across different markets and countries.

A more recent stream of research examines volatility spillover effects in cryptocurrency markets and finds that overall, changes in the price of Bitcoin drive the interconnections between those digital assets. This includes studies analyzing only cryptocurrencies (Moratis 2021; Ampountolas 2022); cryptocurrency and foreign exchange markets (Hsu 2022), Non-Fungible Tokens (NFTs) markets (Wang 2022), Bitcoin and Alternative Coins (altcoin) (Nguyen et al. 2019), Bitcoin, gold and the US Dollar (Dyhrberg 2016), and stablecoin-linked perpetual futures (De Blasis and Webb 2022). Using BEKK-multivariate generalized autoregressive conditional heteroskedasticity (MGARCH) analysis (Katsiampa et al. 2019), recent literature finds evidence of volatility spillover effects among major cryptocurrencies and that changes in Bitcoin prices contribute to return and volatility spillovers among major cryptocurrencies (Koutmos 2018). Interestingly, Smales (2020) finds a single component highly correlated with Bitcoin returns is responsible for a large amount of cryptocurrency return variation. Moreover, there is some evidence of asymmetry in volatility spillovers. For instance, Smales (2021) find evidence of spillovers from Bitcoin and ETH to Tether, but not in reverse. Yi et al. (2018) establishes that large cryptocurrencies are tightly connected to the market and primarily responsible for volatility shocks. Finally, Jarno and Kołodziejczyk (2021), who analyze the average volatility of 20 stablecoins, excluding BUSD, determines important volatility differences between coins in non-volatile periods. For a comprehensive survey on cryptocurrency trading and more broadly blockchain, see Fang et al. (2022) and Xu et al. (2019), respectively.

From all the streams of research related to our study reviewed above, we hypothesize that differences in the way a stablecoin maintains its peg may produce differences in trader behavior and, consequently, in market reactions and contagion effects during turbulent periods. We expect volatility spillover effects between digital assets during periods of market turbulence and our experiment provides a natural opportunity to study the impact of a cryptocurrency market crash. In addition, our data on different stablecoins enable us to test whether our hypothesis that the design of these assets matters. Crucially, this crash was sparked, in part, by the collapse of a stablecoin, presenting perhaps the first natural opportunity to study market reaction, including among other stablecoins, to an important stablecoin failure.

## Institutional details

A stablecoin is understood as a cryptocurrency or token designed to maintain a “stable” peg to another currency, usually the US Dollar, on a one-for-one basis. Although they share the common objective of maintaining a stable peg to the US dollar, stablecoins often differ sharply in the mechanism used to ensure stability by maintaining the peg. Indeed, there is a wide variety of stablecoins with differing designs. According to a US Government report on Stablecoins, depending on its design, a stablecoin can be classified as a security, a commodity, and/or a derivative.^6^

In the United States, most stablecoins are treated as * value that substitutes for currency*,

although this status may differ, with differing treatment even at the State level. Despite the fact they are all attempting to mirror the US dollar on a one-for-one basis, substantial legal, design, and market performance differences remain between major stablecoins. Stablecoins support their pegs to the US dollar via various mechanisms, including cash, treasuries, corporate paper, algorithms, or other cryptocurrencies. For example, on June 8, 2022, Adrienne Harris (2022), Superintendent of the New York State Department of Financial Services, announced a regulation mandating that stablecoins issued by entities licensed by New York State must be fully backed by reserves with a redemption plan approved in advance by the Department of Financial Services, among other requirements. However, not all major stablecoins are in compliance with this regulation. The design and reserve structure of the major stablecoins is summarized in Table 1.

**Table 1:** Comparison of bitcoin and popular stablecoins by backing, market capitalization, and trading volume as of 7:50 a.m. UTC, June 28, 2022

Coin	Asset class	Financial backing structure	Market cap ($)	Volume ($-24 h)
USDT	Stablecoin	Diversified reserves	66,759,936,925	41,133,272,015
USDC	Stablecoin	Cash and US bonds	55,826,655,034	4,330,623,305
BUSD	Stablecoin	$1 for 1	17,439,109,190	5,255,903,027
DAI	Stablecoin	Collateralized debt	6,780,302,604	250,145,391
UST	Stablecoin	Algorithmic design, some cryptocurrency reserves	476,000,027	97,740,154
BTC	Cryptocurrency	N/A	398,452,637,815	21,643,828,415

The question naturally arises as to why stablecoins are used. Lyons and Viswanath-Natraj (2020) argue that Stablecoins sometimes trade at a premium^7^ to the underlying asset they mimic because of the high fees to trade US dollars, the difficulty of using US dollars on cryptocurrency exchanges, and the speed and ease of transferring stablecoins between exchanges. Another reason may be the extreme volatility of some cryptocurrencies. By comparison, the US dollar and the stablecoins that mimic it are generally less volatile.

An article published in Bloomberg by Brown (2021) claims “the real reason people use stablecoins is regulations make it difficult to convert crypto assets to traditional assets. Stablecoins are a creature of regulation in the same sense that money market funds were created in the 1970s to get around government limits on interest that banks could pay retail depositors while the economy was running at double-digit inflation”. The most popular, and liquid, stablecoins include Tether, DAI, TerraUSD, and USDC. Each is considered in the Appendix, with particular emphasis on their design characteristics, differences, and limitations. We then test how these differences in stablecoin designs affect trader behavior, market reactions, and contagion effects during periods of turbulence.

## Methodology

To test the financial contagion effect between stablecoins, we follow the approach proposed in Celık (2012), who presented evidence of contagion during the U.S. subprime crisis via the DCC–GARCH model developed by Engle (2002). The DCC–GARCH model is a class of multivariate GARCH models used to measure conditional covariances and correlations, and thus the interaction between time series. Departing from the methodology in Celık (2012) and considering that the BEKK model developed by Engle and Kroner (1995) is preferred over the DCC–GARCH model (Caporin and McAleer 2012), we assess the existence of contagion effects during the UST collapse by employing the BEKK model.

Assuming that the log returns follow a normal distribution with zero means and variance-covariance matrix $H_t$, we can model the conditional covariances as

1 $$\begin{aligned} H_t=C C^{\prime }+A\left( e_{t-1} e_{t-1}^{\prime }\right) A^{\prime }+B H_{t-1} B^{\prime } \end{aligned}$$

where *C*, *A* and *B* are parameters matrices with *C* being lower triangular.

The BEKK representation in (1) poses some difficulties during the estimation process as the number of parameters is very high when considering many time series. To reduce the parameters, we employ a scalar version of (1) and apply the concept of variance targeting to eliminate the term $CC'$. Thus, the model becomes

$$\begin{aligned} H_t=(1-a-b) {\bar{H}}+a\left( e_{t-1} e_{t-1}^{\prime }\right) +b H_{t-1}, \end{aligned}$$

where ${\bar{H}}=\sum _{t=1}^T e_{t-1} e_{t-1}^{\prime }$ denotes the unconditional covariance matrix estimated from the full sample. In this scalar version, the only parameters are *a* and *b*, subject to *a*, $b > 0$, and $a + b < 1$. These constraints are imposed to keep the process stationary and guarantee the positive definiteness of the covariance matrices.

Once we obtain the conditional covariances, and thus the conditional correlations, we can perform the contagion test as proposed in Celık (2012). The hypothesis is

$$\begin{aligned} H_0: \mu _{\text {pre}}=\mu _{\text {post}}, \end{aligned}$$

where $\mu _{\text {pre}}$ and $\mu _{{post}}$ are the matrices of the means of the conditional correlations from the population during the UST pre-collapse and collapse periods, respectively, with variances $\sigma _{\text {pre}}$ and $\sigma _{\text {post}}$. Considering two samples with sizes $n_{\text {pre}}$ and $n_{\text {post}}$ and the matrices of the means of the conditional correlations computed from the BEKK model, ${\bar{\rho }}_{\text {pre}}$ and ${\bar{\rho }}_{\text{ pre }}$ with variances $s_{\text {pre}}^2=\frac{1}{n_{\text {pre}}-1} \sum _{t=1}^{n_{\text {pre}}}\left( \rho _{\text {pre}}-{\bar{\rho }}_{\text {pre}}\right) ^2$ and $s_{\text {post}}^2=\frac{1}{n_{\text {post}}-1} \sum _{t=1}^{n_{\text {post}}}\left( \rho _{\text {post}}-{\bar{\rho }}_{\text {post}}\right) ^2$, we can compute the t-statistics as

$$\begin{aligned} t=\frac{\left( {\bar{\rho }}_{\text {post}}-{\bar{\rho }}_{\text {pre}}\right) -\left( \mu _{\text {post}}-\mu _{\text {pre}}\right) }{\sqrt{\frac{s^2_{\text {post}}}{n_{\text {post}}}+\frac{s^2_{\text {pre}}}{n_{\text {pre}}}}}, \end{aligned}$$

with degrees of freedom

$$\begin{aligned} v=\frac{\left( \frac{s^2_{\text {post}}}{n_{\text {post}}}+\frac{s^2_{\text {pre}}}{n_{\text {pre}}}\right) ^2}{\frac{\left( \frac{s^2\text {post}}{n_{\text {post}}}\right) ^2}{n_{\text {post}}-1}+\frac{\left( \frac{s^2\text {pre}}{n_{\text {pre}}}\right) ^2}{n_{\text {pre}}-1}}. \end{aligned}$$

When the t-statistic is significantly greater than the critical value, the null hypothesis is rejected, supporting the existence of a contagion effect.

## Data

This study uses proprietary minute-by-minute price transaction data for the most liquid cryptocurrency, Bitcoin (BTC), and the six most liquid stablecoins, namely, Tether (USDT), Binance Coin (BUSD), US Dollar Coin (USDC), Dao Coin (DAI), TerraUSD (UST), and Terra (LUNA), the companion cryptocurrency linked to UST. The sample spans a 40-day period extending from April 20 to May 29, 2022, and covers a symmetrical pre- and post-period of 20 days around the TerrUSD crash between the 9th and the 10th of May 2022. We collect the data from different exchanges and providers, such as Kaiko (for BTC, USDT, USDC, DAI, and UST), and CryptoCompare (for BUSD and LUNA), all supplied by Refinitiv (formerly Thomson Reuters), a London Stock Exchange Group (LSEG) business, and sourced from the Thomson Reuters Tick History (TRTH) database. The final dataset consists of 57,600 price observations of the seven digital assets.

Given that the cryptocurrency market is fragmented with many alternative trading venues, the question naturally arises as to which price series to analyze. We use price data from Refinitiv because it is a weighted average of the prices reported on various exchanges. This decision reduces the observed volatility and magnitude of the price moves in response to the news because price data are essentially smoothed by averaging. However, we believe that this drawback is outweighed by the fact that the smoothed data avoid giving too much weight to transactions on smaller trading venues, thereby presenting a more accurate snapshot of where the price was at any moment in time.

We compute cryptocurrency and stablecoin returns as $ln(P_t /P_{t - 1})$ where $P_t$ is the price of the digital asset at time *t*. According to the literature, determining the cut-off date of a crisis period may not be straightforward (Kaminsky and Schmukler 1999). Consequently, we consider the very beginning of a significant decline in the UST price, which also coincides with the day of the first news-based announcement of the stablecoin potential crash. Therefore, we use midnight of the 10th of May 2022 as the starting point of the collapse period. Finally, we calculate cumulative abnormal returns (CARs) for the purpose of the second analysis. For stablecoins, we assume that the expected return is $E[RS] = 0$ and calculate, before summing, abnormal returns by subtracting the expected return of a stablecoin to its actual return computed as described above. For Bitcoin, instead, we calculate a benchmark BTC return during the first two days of the sample and then subtract this benchmark from the actual BTC return each day in each minute to obtain the abnormal returns before cumulation.

## Results

Table 2 illustrates the descriptive statistics of stablecoin returns during both periods (i.e., pre-collapse in Panel A and UST collapse in Panel B) and for the entire sample (Panel C). To run the analysis, we test whether the returns (and squared returns) are normally distributed via the Jarque–Bera test, whether the null hypothesis that a unit root is present in the returns time-series sample through the augmented Dickey–Fuller test, whether there is heteroskedasticity in the sample distribution with the ARCH model, and finally, the Ljung–Box test for autocorrelations within our data. All statistical tests are consistently significant at the 1% level for all three periods. Panel C also clearly indicates that the assumption made in the methodology section holds because all the returns have approximately zero means. Another noteworthy statistic is that the median is 0 for all the return distributions during every period. As in Celık (2012), all the distributions of returns are leptokurtic, which is a common characteristic of financial market data.

**Table 2:** Descriptive statistics of Stablecoins returns

	BTC	BUSD	DAI	LUNA	UST	USDT	USDC
Panel A: pre-collapse period (20 April 2022–9 May 2022)							
Mean	− 0.0011	0	0	− 0.004	− 0.0009	0	0
Median	0	0	0	0	0	0	0
Max	1.3604	0.01	0.6764	7.5653	5.9968	0.626	1.0925
Min	− 1.2013	− 0.01	− 0.6765	− 4.6747	− 2.8252	− 0.6271	− 0.8492
SD	0.0846	0.0045	0.0263	0.1932	0.0591	0.0183	0.0255
Skewness	0.1752	− 0.0002	0.0545	0.7353	25.7039	0.1591	1.9935
Excess Kurtosis	19.1692	2.0482	60.5877	157.2306	4198.0988	231.8142	466.2319
Jarque–Bera	0.44***	0.05***	4.41***	29.67***	21152.01***	64.49***	260.87***
ADF	− 23.1***	− 40.1***	− 38.4***	− 14.1***	− 3.3**	− 36.5***	− 39.4***
ARCH(1)	1492.1***	211.5***	6866.4***	719.0***	29.2***	7084.0***	8035.1***
ARCH(6)	2656.9***	444.5***	9637.9***	2732.5***	1038.9***	11674.6***	8705.1***
ARCH(12)	2926.4***	602.3***	9748.4***	5020.3***	1880.5***	12069.9***	8736.9***
Q(6)	30.7***	2509.1***	7037.5***	324.5***	1235.3***	6469.0***	8112.6***
Q(12)	64.1***	2522.6***	7049.2***	432.0***	1834.3***	6480.5***	8113.4***
Q$^2$(6)	4823.9***	567.3***	7051.2***	4236.6***	1122.0***	7122.2***	11766.0***
Q$^2$(12)	7139.7***	928.3***	7303.3***	10180.7***	2125.8***	7130.7***	11766.1***
Panel B: collapse period (10 May 2022–29 May 2022)							
Mean	− 0.0001	0	0	− 0.0437	− 0.0118	0	0
Median	0	0	0	0	0	0	0
Max	2.5506	0.1	1.8215	608.336	43.8416	1.7807	6.3763
Min	− 1.8623	− 0.1	− 2.6808	− 607.3849	− 46.106	− 1.7589	− 6.4213
SD	0.1302	0.0137	0.0612	52.9634	1.7118	0.0584	0.097
Skewness	0.8269	0.0016	− 1.7094	− 0.0164	0.2579	− 0.1884	0.5163
Excess Kurtosis	23.612	41.9405	210.6163	108.6154	89.4661	160.844	1418.0227
Jarque–Bera	0.67***	2.11***	53.25***	14.16***	9.61***	31.05***	2412.95***
ADF	− 24.1***	− 34.2***	− 38.1***	− 32.0***	− 22.1***	− 29.8***	− 38.5***
ARCH(1)	1678.3***	4751.9***	686.7***	7409.7***	2721.6***	5390.1***	7088.5***
ARCH(6)	3383.1***	8253.2***	4212.7***	11858.9***	3749.0***	6446.3***	11601.0***
ARCH(12)	3762.7***	9288.2***	5717.0***	12365.3***	4719.2***	7627.5***	12291.3***
Q(6)	21.1***	4910.3***	5034.8***	7721.5***	292.8***	4152.2***	6675.5***
Q(12)	50.0***	4967.9***	5164.5***	7887.4***	336.0***	4457.9***	7158.2***
Q$^2$(6)	6528.1***	23465.1***	4038.3***	40460.8***	5904.1***	10990.2***	7094.9***
Q$^2$(12)	10228.6***	45786.2***	7064.8***	74407.5***	9964.4***	19652.8***	8124.4***
Panel C: entire period (20 April 2022–29 May 2022)							
Mean	− 0.0006	0	0	− 0.0239	− 0.0064	0	0
Median	0	0	0	0	0	0	0
Max	2.5506	0.1	1.8215	608.336	43.8416	1.7807	6.3763
Min	− 1.8623	− 0.1	− 2.6808	− 607.3849	− 46.106	− 1.7589	− 6.4213
SD	0.1098	0.0102	0.0471	37.451	1.2111	0.0433	0.0709
Skewness	0.7357	0.0021	− 1.8707	− 0.0248	0.3521	− 0.2256	0.7069
Excess Kurtosis	27.246	70.5863	304.6165	220.2247	181.4897	272.5424	2488.7769
Jarque–Bera	1.79***	11.96***	222.73***	116.40***	79.05***	178.27***	14865.63***
ADF	− 33.0***	− 44.1***	− 49.4***	− 41.1***	− 29.1***	− 39.9***	− 49.2***
ARCH(1)	3424.5***	9740.3***	1456.8***	14949.5***	5578.0***	10893.0***	14188.9***
ARCH(6)	6763.1***	16830.6***	8361.1***	23865.9***	7701.9***	12990.2***	23185.7***
ARCH(12)	7490.7***	18902.5***	11344.4***	24880.7***	9667.5***	15311.5***	24562.2***
Q(6)	26.1***	9233.3***	10516.0***	15441.7***	581.3***	8589.3***	13293.0***
Q(12)	70.6***	9327.7***	10710.5***	15773.4***	667.0***	9097.4***	14135.8***
Q$^2$(6)	13175.9***	48299.2***	8106.1***	81691.3***	12327.5***	22026.3***	14202.6***
Q$^2$(12)	20655.8***	94295.5***	14136.6***	150341.0***	20902.1***	39178.6***	16264.7***

The table shows the descriptive statistics for pre-collapse, collapse and the entire period. Jarque–Bera represents the test statistics from the normality test (expressed in $\times 10^6$). *ADF* represents the augmented Dickey–Fuller test. *ARCH*(6) and *ARCH*(12) correspond to the test statistics from the ARCH test with 6 and 12 lags respectively. *Q*(6), *Q*(12) and $Q^2$(6), $Q^2$(12) represent the test statistics from the Ljung–Box test for serial correlation in returns and squared returns with 6 and 12 lags respectively. ***Indicates the rejection of the null hypothesis at the 1% significance level

**Fig. 1:**
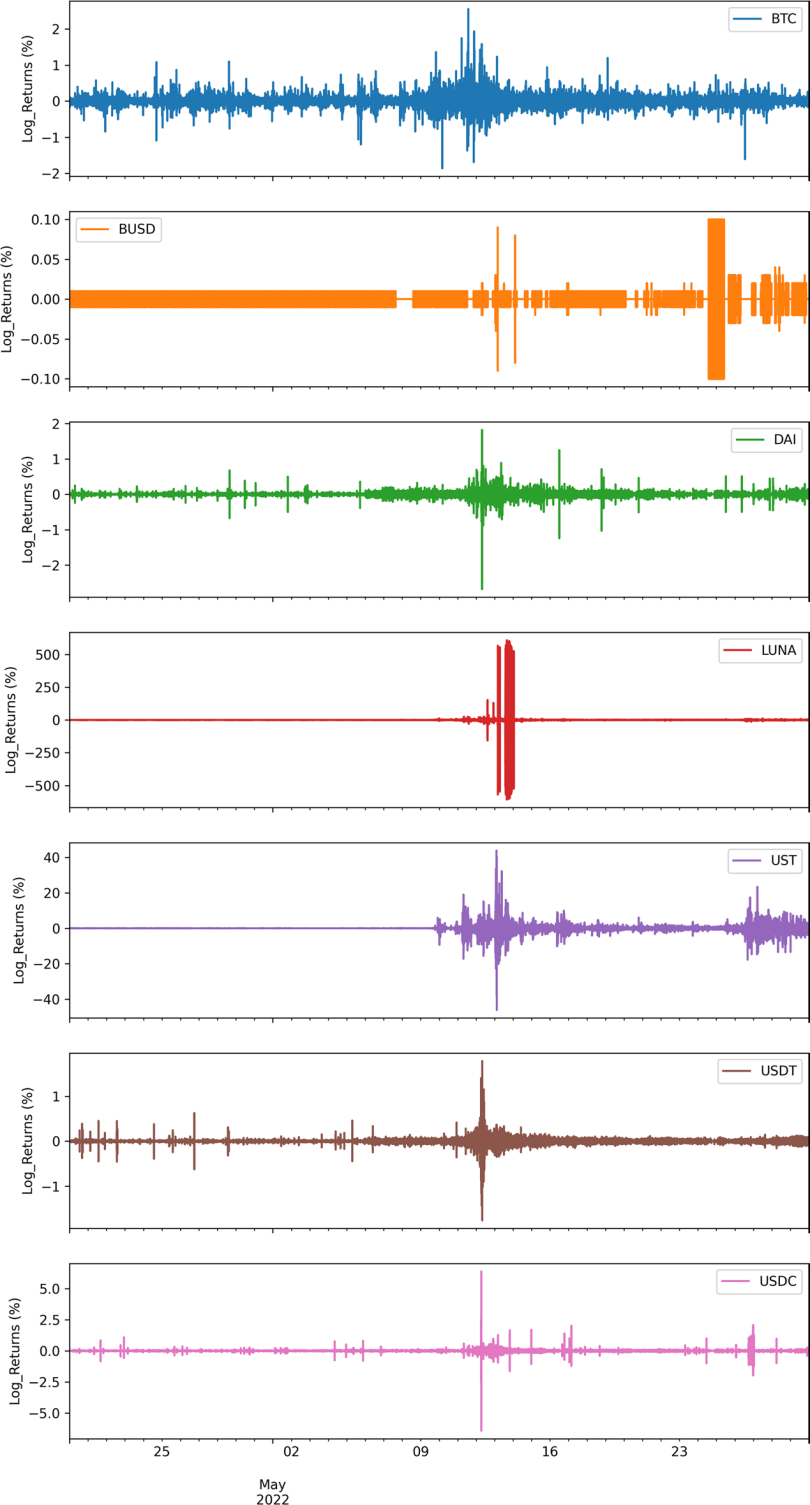
Stationary returns

Figure 1 illustrates the stationary returns of all the cryptocurrencies examined over the sample period. An anomaly is evident on the right-hand side of each chart. Namely, the charts show that abnormal returns occurred after UST started to collapse. Interestingly, the day on which the highest return occurred for UST and LUNA differs from the spike in USDT, USDC and DAI. This may signal an information cascade effect. Table 3 presents the dynamic conditional correlation matrices between all cryptocurrencies during the pre-collapse (Panel A) and collapse (Panel B) periods.

**Table 3:** BEKK dynamic conditional correlation matrices

	BTC	BUSD	DAI	LUNA	UST	USDT	USDC
Panel A: pre-collapse period (20 April 2022–9 May 2022)							
BTC	1	0.0881	− 0.0024	0.5448	− 0.0146	− 0.0053	0.0024
BUSD	0.0881	1	0.0026	0.0406	− 0.0074	0.0041	0.0040
DAI	− 0.0024	0.0026	1	− 0.0024	0.0038	0.0115	− 0.0016
LUNA	0.5448	0.0406	− 0.0024	1	0.0997	0.0012	− 0.0048
UST	− 0.0146	− 0.0074	0.0038	0.0997	1	− 0.0040	− 0.0039
USDT	− 0.0053	0.0041	0.0115	0.0012	− 0.0040	1	0.0172
USDC	0.0024	0.0040	− 0.0016	− 0.0048	− 0.0039	0.0172	1
Panel B: collapse period (10 May 2022–29 May 2022)							
BTC	1	0.0148	− 0.0078	0.0059	− 0.0041	0.0024	0.0056
BUSD	0.0148	1	0.0008	− 0.0027	0.0013	− 0.0035	− 0.0118
DAI	− 0.0078	0.0008	1	0.0059	0.0271	0.0239	0.0027
LUNA	0.0059	− 0.0027	0.0059	1	− 0.0055	0.0119	− 0.0055
UST	− 0.0041	0.0013	0.0271	− 0.0055	1	− 0.0041	0.0017
USDT	0.0024	− 0.0035	0.0239	0.0119	− 0.0041	1	− 0.0140
USDC	0.0056	− 0.0118	0.0027	− 0.0055	0.0017	− 0.0140	1

Pre-collapse period is from 20.04.2022 to 09.05.2022. Collapse period is from 10.05.2022 to 29.05.2022

**Fig. 2:**
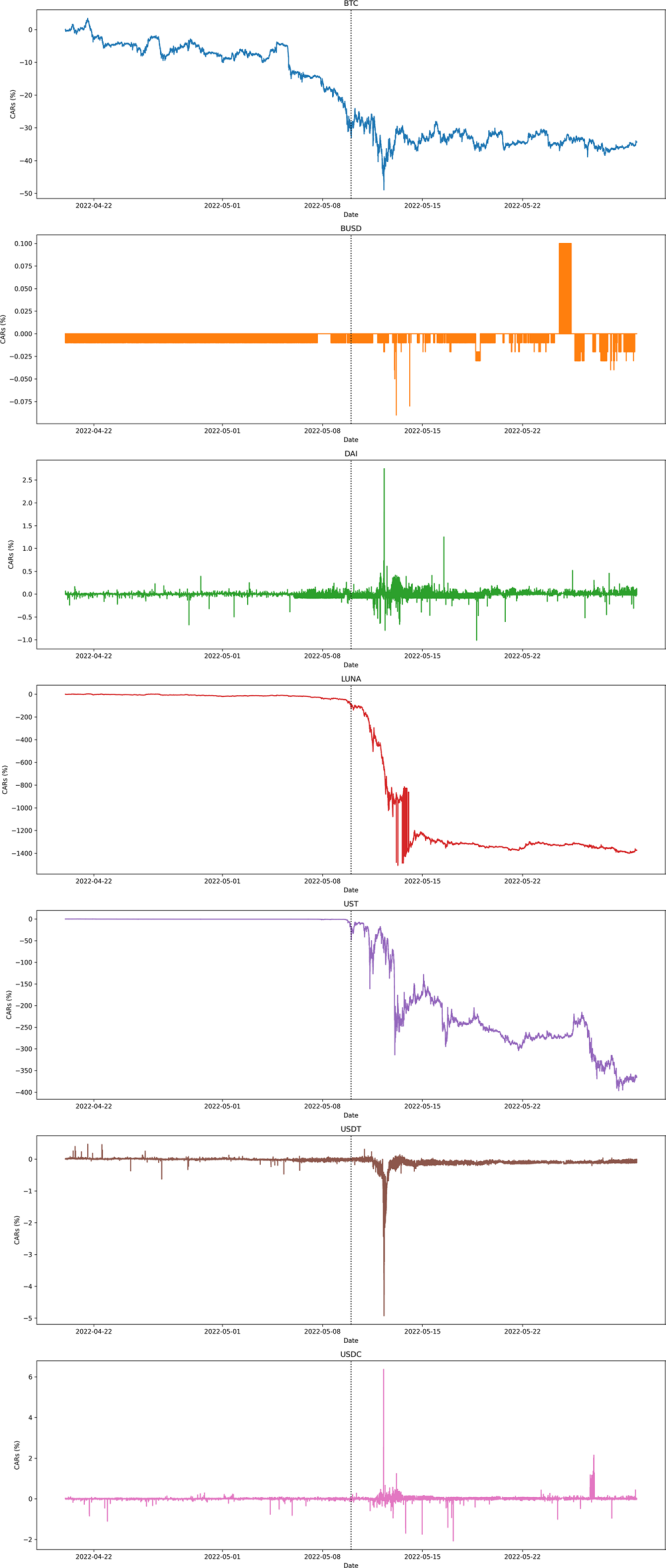
Cumulative abnormal returns

**Fig. 3:**
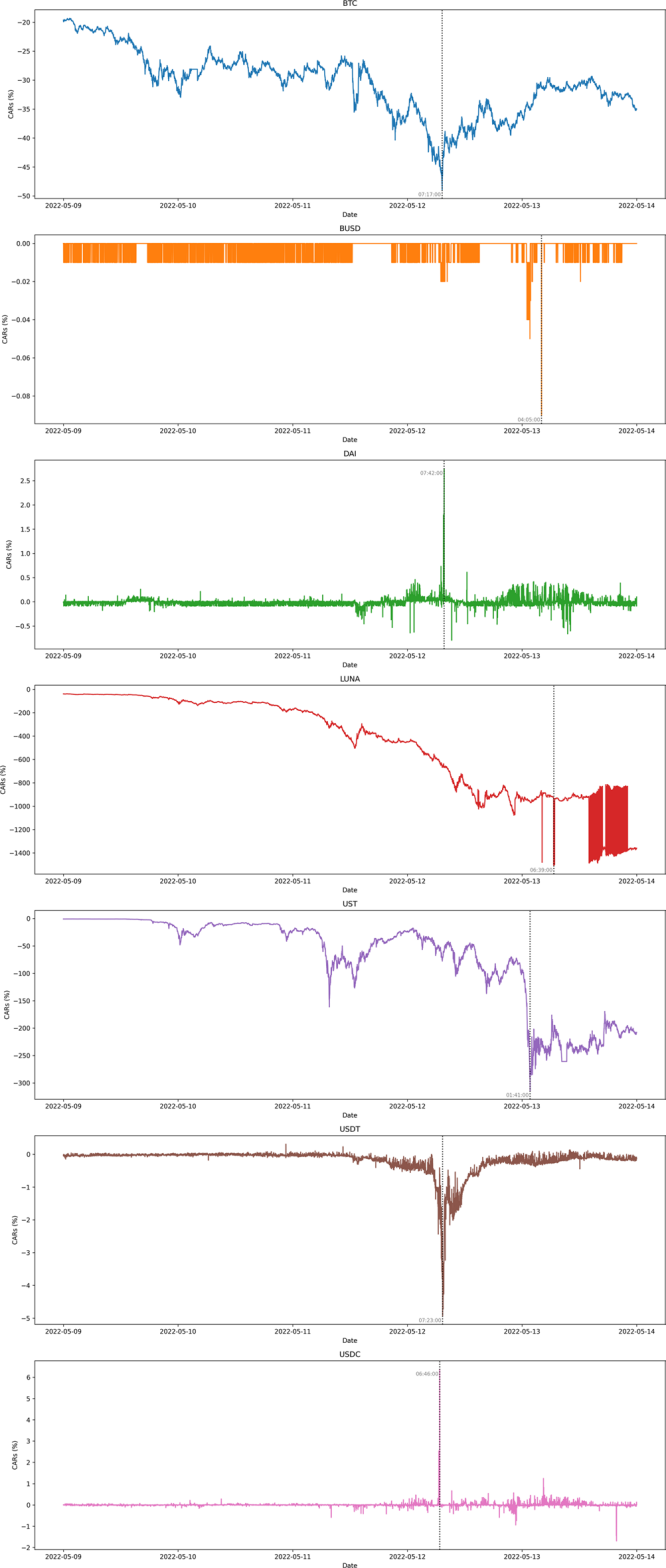
Cumulative abnormal returns zoomed-in

Of course, a stablecoin should ideally have zero cumulative returns as it should maintain a precise peg with the US dollar. The results confirm that traders are responsive to the underlying design of the cryptocurrency, and the underlying design itself affects trader activity. For example, BUSD,—which is backed dollar-for-dollar with cash in US banks and regulated in New York, was the beneficiary of a flight to safety during the collapse period. Figure 2 illustrates the cumulative abnormal returns (CARs) of the digital assets analyzed over the entire sample period, whereas Fig. 3 presents the CARs during the period of greater price reactions between May 9 and 13, and 2022. The zoomed-in version of the CARs also outlines the information cascade that started from the UST and, simultaneously, LUNA, whose underlying is based on UST, and then spread to USDT a couple of days later on May 12, which spilled over to USDC and DAI almost instantaneously before bouncing back to UST and LUNA and also slightly affecting BUSD. There was a clear market reaction at the event of the USDT decline that precipitated a sharp increase of approximately $6\%$ in USDC CARs within a couple of hours. One hour later, USDT reached its all-time low with a cumulative abnormal return of $-5\%$, causing a simultaneous spike in DAI (a positive cumulative abnormal return of $3\%$). Only a day later, UST collapsed entirely to a handful of cents, with a decline of $9x\%$, triggering a slight de-peg of a negative CAR near $-0.1\%$ even in BUSD. Bitcoin CARs dropped to $-50\%$, while overall UST and LUNA crashed with a magnitude of approximately $-300\%$ and $-1500\%$ cumulative abnormal returns, respectively. A non-technical analysis reveals a two-day delay in market events happening on centralized exchanges that could have been predicted by examining market activities in decentralized liquidity pools (Melachrinos 2022). However, this topic remains a topic for future research.

**Fig. 4:**
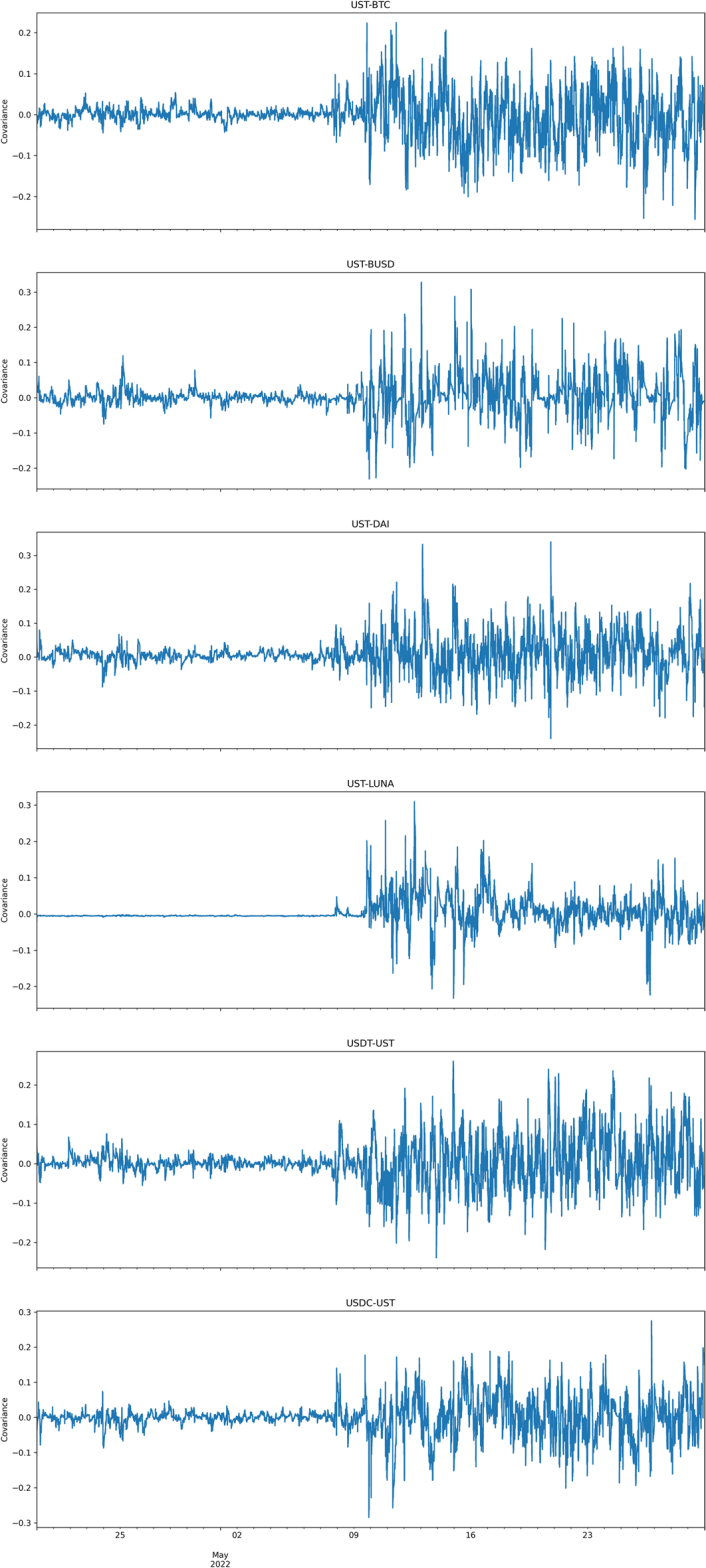
BEKK covariances

Table 4 presents the dynamic conditional covariance estimates of the BEKK-GARCH model and the relative *t*-test statistics on the existence of contagion. Evidence indicates that the TerraUSD collapse precipitated a spillover effect across all the major stablecoins analyzed, in addition to bitcoin. All tests are statistically significant at the 1% level, supporting the existence of contagion effects. Figure 4 illustrates these dynamic conditional covariances plotted throughout the sample period. The right-hand side of all charts clearly presents an evident movement in the stationary covariances, meaning that after the UST collapse, all other digital assets experienced a significant price movement caused by a spillover effect in the period after the 9th of May 2022.

**Table 4:** BEKK dynamic conditional covariance coefficients and contagion effect tests

	Mean	Variance	T-statistic
Pre-collapse BEKK covariance UST_BTC	0.0013	0.0003	24.64***
Collapse BEKK covariance UST_BTC	− 0.0084	0.0042	
Pre-collapse BEKK covariance UST_BUSD	− 0.0009	0.0004	− 24.71***
Collapse BEKK covariance UST_BUSD	0.0087	0.0040	
Pre-collapse BEKK covariance UST_DAI	0.0052	0.0003	− 23.96***
Collapse BEKK covariance UST_DAI	0.0141	0.0037	
Pre-collapse BEKK covariance UST_LUNA	− 0.0038	0.0001	− 44.15***
Collapse BEKK covariance UST_LUNA	0.0091	0.0023	
Pre-collapse BEKK covariance UST_USDT	0.0009	0.0004	− 14.78***
Collapse BEKK covariance UST_USDT	0.0072	0.0048	
Pre-collapse BKK covariance UST_USDC	− 0.0004	0.0004	− 3.84***
Collapse BEKK covariance UST_USDC	0.0011	0.0041	

Pre-collapse period is from 20.04.2022 to 09.05.2022. Collapse period is from 10.05.2022 to 29.05.2022. Entire period is from 20.04.2022 to 29.05.2022. ***Indicates the significance level at 1%

These results indicate a statistically significant level of contagion between UST and other stablecoins. This suggests that the UST collapse was responsible for the broader dislocation and contagion in the stablecoin market in May 2022. The differential behavior of stablecoins and cryptocurrency assets suggest that herding among traders is a likely cause of these market results. Although UST caused the initial crash in the stablecoin market, it was not until a collapse in USDT on May 12, 2022, led to an even further, and final blow to UST. Moreover, the duration of the impact was uneven across cryptocurrencies. The impact did not persist for BUSD, but Tether continued to deviate from its dollar peg until the 19th of July 2022, albeit with a deviation far smaller than that experienced during the initial reaction to the news. This demonstrates how smaller market players can cause financial contagion, which infects larger players, finally feeding back to the market as a whole.

## Conclusion

To date, the collapse of Terra and LUNA is the largest stablecoin collapse. It is also the largest collapse of an algorithmic stablecoin and cryptocurrency pair, which itself is a unique design among major cryptocurrencies. These factors alone make the Terra and LUNA collapse worthy of investigation. However, this collapse has broader implications for stablecoins and cryptocurrencies as a whole. This study examined the contagion effects across cryptocurrency assets and how stablecoin design affects stablecoin reaction to price shocks in the period surrounding the Terra and LUNA crashes. This illustrates in vivid detail how stablecoin design may affect the price stability of cryptocurrency assets.

We examined differences in the magnitude, direction, and duration of their responses using a multivariate BEKK model over a sample period of 40 days surrounding the crash on the 9th of May 2022. We found evidence of a contagion effect across all cryptocurrencies analyzed, with potential signs of herding behavior by traders after an information cascade. Traders voted with their feet, buying stablecoins with safer designs such as BUSD, which is backed by $1 for 1 with cash in a US Bank. USDC also rose above $1, whereas DAI fluctuated around $1. This demand was so high that USDC and BUSD reached 1.01 on at least some exchanges, indicating that traders were willing to pay extra for a flight to safety.

Paying $1.01 for a $1 asset is uneconomic, suggesting major concerns about stability and potentially even the survival of other cryptocurrencies or stablecoins. It also illustrates the costs of switching from bitcoins into US dollars. This fear was exhibited most prominently in the price action of UST, which faced a near-total collapse, and also in Tether, which traded as low as 95 cents on some exchanges. The fact that the obvious arbitrage opportunity to buy a $1 asset for 95 cents was not immediately eliminated suggests that fears of a broader collapse were widespread.

Herd behavior may help explain how traders seek perceived safety, overpaying for “safe” stablecoins while selling stablecoins deemed unsafe during turbulent periods. Stablecoin developers, exchanges, and regulators should consider the results of this study to design more robust systems to prevent other scenarios in which a stablecoin de-pegging process spills over negative effects across other digital assets and deteriorates the market by allowing flash crashes. The fact that Tether, the most popular stablecoin by market capitalization and volume, which generally trades for $1 was able to be purchased for 95 cents speaks to the depth of the market uncertainty.

Future research may compare the institutional and algorithmic designs of various stablecoins to further investigate market reactions to various design structures. Should the data become available, a comparison of the reserve structure and quality of the major stablecoins would create interesting research opportunities. Our research suggests that market participants can accurately discriminate among stablecoins in terms of their safety during a crash, but may continue to trade coins with larger market capitalization, even if they may be riskier, during less volatile times. A subsequent future research avenue is the potential prediction of cryptocurrency market crises using decentralized exchange (DEXs) liquidity pool data, which could potentially shed more light on the interconnections between decentralized and centralized markets and the reasons behind traders’ herding behaviors during turbulent periods. In line with Sebastião and Godinho (2021), this may be achieved using machine learning techniques.

One limitation of this study is that its findings cannot be extended to normal periods of more tranquil markets. Despite its poor performance during this crisis, Tether remains an important stablecoin. Therefore, future studies may attempt to ascertain the continued popularity, demonstrated via market capitalization and liquidity measures, and the price movements of less regulated stablecoins outside crisis periods.

## Data Availability

The data that support the findings of this study are available from Refinitiv but restrictions apply to the availability of these data, which were used under license for the current study, and so are not publicly available. Data are however available from the authors upon reasonable request and with permission of Refinitiv and Rozetta.

## Appendix

### Tether

Tether is the largest stablecoin by market capitalization and most popular by trade volume.^8^ Tether’s website^9^ claims its reserves are 85.64% “Cash and cash equivalents and other short-term deposits and commercial paper”. With another 6.02% in “Other Investments” which includes digital tokens. The Financial Times^10^ and Bloomberg^11^ have both published articles questioning the makeup of Tether’s commercial paper holdings, with the articles discussing speculation that some of the assets may be marked down “Chinese or Asian”^12^ commercial paper, a charge Tether has denied, amidst worries over the creditworthiness of some mainland Chinese firms.^13^

Restrictions on the redemption of Tether exist. A 150 USDT verification fee is required to set up an account, while withdrawal fees of.01% or $1000 (whichever is greater) apply to any withdrawal. $100,000 is the current minimum withdrawal size.^14^ Most U.S. persons or entities cannot withdraw fiat currency with the service. As a result, the redemption ability of Tether only approaches a $1 for 1 ratio at very high withdrawal sizes.^15^

Tether’s price has diverged from $1 on multiple occasions. For example, on March 12, 2020, it traded at $1.05. On March 17, 2021, it traded below 98 cents. Those examples are indicative and not exhaustive. On February 17, 2021, Tether settled a lawsuit from New York Attorney General, Letitia James, who said “Tether’s claims that its virtual currency was fully backed by US dollars at all times was a lie”. Tether agreed to pay $18.5 million and end trading with New York residents and entities.

The incentive structure of coins like Tether has also come into question. Economist Tyler Cowen has suggested that Stablecoin issuers have incentives to print stablecoins in excess of the amount that reserves would directly support. In a thought experiment, he argues; “If the price of your coin stays at $1, fine, you come out ahead. If the price declines in proportion to the new and higher risk, you as an issuer still have broken even”.^16^

### DAI

DAI is a stablecoin on the Ethereum blockchain which maintains its peg to the dollar using a series of smart contracts and the Target Rate Feedback Mechanism (TRFM). If Dai falls below $1, the TRFM will increase, incentivising the market to push the price up. Dai tokens are created through borrowing. Users lock collateral on the blockchain, and they receive DAI in the amount of their locked collateral. The DAI is burned when this collateral is repaid.^17^

DAI was created by MakerDAO, a decentralized organization. DAI itself is also decentralized, thus, anyone can create DAI using accepted forms of collateral. Dai can be shut down in a semi-democratic process known as Global Settlement.

Collateral is not necessarily exchanged for DAI at a one-to-one basis. On April 1, 2022, the MakerDAO twitter account noted a user with $30,000 of collateral could create at most, 20,689 DAI. IDAI has historically been collateralized only with cryptocurrency assets, but in June 2022 participants voted to begin investing $500 million in US Treasury bills.^18^

On March 13, 2020, amidst financial uncertainty during the early stages of the COVID-19 pandemic, DAI traded at $1.09.^19^

### TerraUSD

TerraUSD was an algorithmically balanced stablecoin, whose design was supposed to use market incentives to maintain parity with the dollar. TerraUSD was linked to Luna, with the names of these currencies analogizing their relationship to that of the earth and the moon. Beginning on May 9, 2022, a fall in UST led to a collapse of the peg between UST and the dollar. The cryptocurrency has never fully recovered, and as of July 5, 2022, UST trades at around 6 cents, after falling to under 1 cent in June 2022.

TerraUSD was theoretically pegged to $1 and supposedly balanced by the expanding or contracting supply of LUNA. When TerraUSD traded below the peg, the protocol incentivized users to “burn” (destroy) TerraUSD and “mint” (create) Luna, balancing the prices.^20^ Every time a new UST was created, $1 of LUNA was “burned” on the ”Terra” Blockchain.

Investopedia noted^21^ “The Terra protocol maintains the price of the Terra stablecoin by ensuring that the supply and demand for it are always balanced. This is achieved by using LUNA as the variable counterweight to the TerraUSD stablecoin”.

Consumers lending UST were offered a nearly 20% interest rate.^22^ Binance marketed this as a “safe and happy” investment opportunity.^23^ Terra founder Do Kwan told Bloomberg that “it’s actually not unnatural for currencies of growing economies to offer higher interest rates than those of mature, stable economies”.^24^ By contrast, USDC deposits offered interest rates of 3.5$-$5.5%, while US dollar deposits at major American banks earned less than 1%. The interest payments came from TerraUSD’s reserves, causing some trepidation amongst investors. It appears likely that the high-interest rate was necessary to keep up demand for UST tokens. Without this demand, the peg could not survive.

An entity known as Luna Foundation Guard-which was once the world’s second-largest known holder of Bitcoin-backed Terra with reserves denominated in cryptocurrency. These reserves primarily included around 80,000 Bitcoin (worth around $2.4 billion on May 7, 2022) as well as approximately $65 million in Avalanche, as well as $12 million in “Binance tokens”.^25^ Nearly all of the Bitcoin reserves were depleted in an apparent effort to maintain the peg.

Several market observers were sceptical about the stability of TerraUSD. Galois Capital called LUNA “doomed to fail” and a “confidence game” around two months before TerraUSD’s and LUNA’s collapse.^26^

On May 25, 2022, Vitalik Buterin, co-creator of Ethereum, argued that stablecoins with the general algorithmic design of UST can become “extremely fragile” if the activity of the asset their price depends on (in this case, LUNA) drops significantly. Because TerraUSD required active trading and value in Luna to balance its own prices, weakness in one currency could lead to problems in the other.

On May 15, 2022, LUNA had a price of $.004173 but an incredible 24-hour volume of $15.92 Billion, showing a dramatic surge of trading activity as the coin collapsed. A month earlier LUNA traded at $84.5 with a 24-hour volume of 2.4 billion.^27^

Months prior to the collapse of TerraUSD, the Securities and Exchange Commission (SEC) filed a subpoena enforcement action against TerraUSD form Labs and Do Kwon relating to the Mirror Protocol, a Decentralized Finance (DeFi) protocol that allowed the creation and trading of digital assets that “mirrored” the prices of securities.^28^

UST had broken the buck before, but never in such a dramatic, sustained fashion. On 12/30/2020 UST reached 85 cents on the dollar before recovering to.9973 the next day. On 1/31/2021 it reached 1.04. On 5/23/2021 it traded at 94 cents on the dollar.^29^ Despite these sharp fluctuations, UST generally traded at just over $1, suggesting some degree of market faith in the project.

What motivated the creation of TerraUSD? Basically, the creators of UST wanted to establish privately issued money. What were the problems that TerraUSD was trying to solve? Essentially, the founders of TerraUSD were trying to facilitate the use of cryptocurrencies as a medium of exchange and store of value or money by eliminating their volatility. They were also trying to facilitate the adoption of UST as a medium of exchange and enlarge the network of users by incentivizing its use. TerraUSD is essentially a pegged currency. If the price falls below its pegged value, then the money supply is reduced and if its price is above its pegged value then the money supply is increased. The plan was to have a companion cryptocurrency, Luna, that acts as collateral and supports the stablecoin. The plan was to encourage arbitrageurs to act to exploit any price discrepancies from the pegged value.

### USDC

USDC was developed by the Center consortium, a partnership between US-based cryptocurrency exchange Coinbase, and US-based peer-to-peer payments company, Circle. In an article published on 13, June 2022, the CFO of Circle wrote that around 80% of USDC reserves are short-dated US Treasuries and around 20% cash.^30^

Circle, the entity which co-founded USDC, “is regulated as a licensed money transmitter under US state law”.^31^ Short monthly “Reserve Account Reports” are available online, with attestations that the “total fair value of US Dollar denominated assets held on behalf of USDC holders is at least” equal to the value of all USDC in circulation. Audits take place yearly as part of Circle’s financial statements.^32^

Underscoring the differing acceptance of stablecoins in the traditional financial industry, on 29 March 2021, Visa announced a pilot program allowing payment settlements with USDC.^33^

## Abbreviations

UST: TerraUSD stablecoin
LUNA: Terra token
BEKK: Baba, Engle, Kraft and Kroner’s multivariate model
US: United States of America
UTC: Coordinated universal time
BUSD: Binance USD stablecoin
USDC: USD dollar coin
DAI: MakerDAO coin
DEXs: Decentralized exchanges
NFTs: Non-fungible tokens
Altcoin: Alternatives coins
DCC: Dynamic conditional correlations
ARCH: Autoregressive conditionally heteroskedasticity
GARCH: Generalized ARCH
USDT: Tether stablecoin
LSEG: London stock exchange group
TRTH: Thomson Reuters Tick History database
CARs: Cumulative abnormal returns
TRFM: Target rate feedback mechanism
SEC: Securities and exchange commission
DeFi: Decentralized finance
DEXs: Decentralized exchanges

## Footnotes

1 Tether’s July 27, 2022, volume at 8:40 a.m. UTC was $45 billion, compared to Bitcoin and Ethereum’s volume of 26 billion and 17 billion, respectively. For another data point showing the same phenomenon consult the chart later in the paper.

2 There is also evidence that an unknown user or entity may have used Tether to inflate the price of Bitcoin (Griffin and Shams 2020).

3 TerraUSD was a stablecoin traded under the symbol UST. It has commonly been referred to as Terra. A related coin traded under the symbol LUNA was technically named Terra. To ease confusion, and to match popular parlance, we refer to the stablecoin as UST or TerraUSD while we refer to the related coin using only its ticker, LUNA.

4 please find information source at https://www.coindesk.com/markets/2022/05/15/the-collapse-of-terra-was-devastating-but-there-is-still-hope-for-crypto/. Accessed July 26, 2022.

5 See Kacperczyk and Schnabl (2010) as well as pp. 24–25 of Pozsar et al. (2013).

6 Further discussion of the designs of various stablecoins is available in the appendix.

7 See, e.g., Frino et al. (2022) for a better understanding of premiums and discounts as well as their market impact.
